# Supplementary material for: Stress diminishes outcome but enhances response representations during instrumental learning
Source: eLife. 2022 Jul 18;11:e67517. doi: 10.7554/eLife.67517 (PMC9355560; doi:10.7554/eLife.67517)
Supplement: Supplementary file 1. — (a) Classification results (block × group interaction) block for alternatively grouped data. (b) Classification results on outcome representations for N=53. (c) Control variables: subjective mood, depressive mood, anxiety, and chronic stress. (d) Eye-tracking data: mean number of blinks and saccades across time, for Shigh and Slow trials (N=51). [file elife-67517-supp1.docx]

**Supplementary File 1A.** Classification results (block × group interaction) block for alternatively grouped data.

|  | O representation during S | O representation during R | R representation  during S |
| --- | --- | --- | --- |
| *Two blocks (288 trials per block)* |  |  |  |
| F | 3.87 | 8.81 | 17.48 |
| df_1,_ df_2_ | 1, 39 | 1, 39 | 1, 49 |
| P | 0.056 | 0.005** | < 0.001*** |
| ƞ_p_² | 0.090 | 0.184 | 0.263 |
| 95% CI | 0 to 0.277 | 0.019 to 0.381 | 0.075 to 0.437 |
| *Three blocks*  *(192 per block)* |  |  |  |
| F | 4.39 | 3.38 | 9.41 |
| df_1,_ df_2_ | 2, 78 | 2, 78 | 2, 98 |
| P | 0.016* | 0.039* | < 0.001*** |
| ƞ_p_² | 0.101 | 0.080 | 0.161 |
| 95% CI | 0.003 to 0.224 | 0 to 0.196 | 0.042 to 0.281 |
| *Four blocks (144 trials per block)* |  |  |  |
| F | 2.77 | 2.99 | 5.82 |
| P | 0.045* | 0.034* | < 0.001*** |
| df_1,_ df_2_ | 3, 117 | 3, 117 | 3, 147 |
| ƞ_p_² | 0.066 | 0.071 | 0.106 |
| 95% CI | 0 to 0.149 | 0 to 0.156 | 0.021 to 0.192 |
| *Six blocks*  *(96 trials per block)* |  |  |  |
| F | 1.91 | 1.86 | 4.73 |
| df_1,_ df_2_ | 5, 195 | 5, 195 | 5, 245 |
| P | 0.094 | 0.103 | < 0.001*** |
| ƞ_p_² | 0.047 | 0.446 | 0.088 |
| 95% CI | 0 to 0.093 | 0 to 0.091 | 0.020 to 0.145 |
| *Twelve blocks*  *(96 trials per block)* |  |  |  |
| F | 1.42 | 1.23 | 2.40 |
| df_1,_ df_2_ | 11, 429 | 11, 429 | 11, 539 |
| P | 0.162 | 0.273 | 0.007** |
| ƞ_p_² | 0.035 | 0.031 | 0.047 |
| 95% CI | 0 to 0.049 | 0 to 0.042 | 0.004 to 0.066 |

*** P < 0.001, ** P < 0.01, * P < 0.05.

**Supplementary File 1B.** Classification results on outcome representations for N = 53

|  | P |
| --- | --- |
| *O representation during S* |  |
| Block × group interaction | 0.005** |
| First six vs. last six blocks (stress group) | < 0.001*** |
| First six vs. last six blocks (control group) | 0.199 |
| Stress vs. control for the last six blocks^1^ | 0.020* |
| Stress vs. control for all lower training intensity^1^ | 1 |
| Correlations with responses to devalued actions during Dev O^high^ blocks | 0.068 |
| *O representation during R* |  |
| Block × group interaction | 0.013* |
| First six vs. last six blocks (stress group) | 0.109 |
| First six vs. last six blocks (control group) | 0.147 |
| Stress vs. control for the last six blocks^1^ | 0.032* |
| Stress vs. control for all lower training intensity^1^ | > 0.420 |

*** P < 0.001, ** P < 0.01, * P < 0.05; ^1^ P values are Bonferroni corrected (vs. each training intensity).

**Supplementary Files 1C.** Control variables: subjective mood, depressive mood, anxiety and chronic stress.

|  | Control | | Stress | |
| --- | --- | --- | --- | --- |
|  | M | SEM | M | SEM |
| *MDBF scales* |  |  |  |  |
| Elevated vs. depressed mood | 17.54 | 0.35 | 17.80 | 0.30 |
| Sleepiness vs. wakefulness | 15.43 | 0.61 | 14.43 | 0.56 |
| Calmness vs. restlessness | 17.21 | 0.50 | 16.73 | 0.44 |
| *BDI* | 8.79 | 1.27 | 6.90 | 1.23 |
| *STAI scales* |  |  |  |  |
| State anxiety | 34.07 | 1.41 | 35.07 | 1.76 |
| Trait anxiety | 35.71 | 1.73 | 36.27 | 1.64 |
| *TICS scales* |  |  |  |  |
| Work overload | 18.36 | 1.12 | 18.43 | 1.19 |
| Social overload | 13.61 | 0.95 | 12.29 | 0.79 |
| Performance pressure | 21.64 | 1.41 | 22.68 | 1.13 |
| Work discontent | 18.32 | 0.96 | 17.96 | 1.22 |
| Excessive workload | 11.61 | 0.70 | 12.43 | 0.92 |
| Lack of social recognition | 8.82 | 0.54 | 8.21 | 0.47 |
| Social tension | 10.71 | 0.78 | 11.32 | 0.67 |
| Social isolation | 13.68 | 0.97 | 13.61 | 0.99 |
| Chronic worrying | 8.54 | 0.54 | 8.32 | 0.59 |
| TICS screening scale | 25.29 | 1.49 | 25.07 | 1.50 |

MDBF, Multidimensional Mood Questionnaire; BDI, Beck Depression Inventory;

STAI, State-Trait Anxiety Inventory; TICS, Trier Inventory of Chronic Stress.

**Supplementary File 1D.** Eye tracking data: mean number of blinks and saccades across time, for S^high^ and S^low^ trials (N = 51).

|  | NoDev | | | | Dev O^high^ | | | | | | | | Dev O^low^ | | | | | | |  |
| --- | --- | --- | --- | --- | --- | --- | --- | --- | --- | --- | --- | --- | --- | --- | --- | --- | --- | --- | --- | --- |
|  | S^high^ | | S^low^ | | S^high^ | | | | S^low^ | | | | S^high^ | | | S^low^ | | | |  |
|  | M | SEM | M | SEM | M | | SEM | | M | | SEM | | M | SEM | | M | | SEM | |  |
| *Mean blinks* |  |  |  |  |  | |  | |  | |  | |  |  | |  | |  | |  |
| Control | 5.20 | 1.13 | 5.28 | 1.18 | 5.67 | | 1.26 | | 5.55 | | 1.30 | | 5.71 | 1.18 | | 6.01 | | 1.30 | |  |
| Stress | 5.13 | 0.79 | 4.90 | 0.80 | 5.17 | | 0.78 | | 5.17 | | 0.88 | | 6.53 | 1.08 | | 6.08 | | 0.97 | |  |
| *Mean saccades* |  |  |  |  |  | |  | |  | |  | |  |  | |  | |  | |  |
| Control | 24.61 | 2.51 | 24.93 | 2.40 | 24.63 | 2.24 | | 23.75 | | 2.24 | | 24.31 | | | 1.98 | | 23.92 | | 2.21 | |
| Stress | 23.36 | 1.56 | 22.36 | 1.64 | 23.06 | 1.74 | | 21.47 | | 1.71 | | 24.17 | | | 1.71 | | 23.71 | | 1.91 | |
