# Supplementary material for: Stress diminishes outcome but enhances response representations during instrumental learning
Source: eLife. 2022 Jul 18;11:e67517. doi: 10.7554/eLife.67517 (PMC9355560; doi:10.7554/eLife.67517)
Supplement: Transparent reporting form [file elife-67517-transrepform1.pdf]

1st Floor
24 Hills Road
Cambridge CB2 1JP, UK

P 01223 855340
W elifesciences.org
T @elife

eLife's transparent reporting form

We encourage authors to provide detailed information *within their submission* to facilitate the interpretation and replication of experiments. Authors can upload supporting documentation to indicate the use of appropriate reporting guidelines for health-related research (see EQUATOR Network), life science research (see the BioSharing Information Resource), or the ARRIVE guidelines for reporting work involving animal research. Where applicable, authors should refer to any relevant reporting standards documents in this form.

If you have any questions, please consult our Journal Policies and/or contact us: editorial@elifesciences.org.

Sample-size estimation

- You should state whether an appropriate sample size was computed when the study was being designed
- You should state the statistical method of sample size computation and any required assumptions
- If no explicit power analysis was used, you should describe how you decided what sample (replicate) size (number) to use

Please outline where this information can be found within the submission (e.g., sections or figure legends), or explain why this information doesn't apply to your submission:

The chosen sample size is based on previous studies on stress and the balance of multiple forms of learning in our lab as well as a-priori power calculation using G*POWER 3 suggesting that the sample size would be sufficient to reveal a medium-sized effect in a mixed-design ANOVA with a power of .80. This information can be found in the 'Participants and design' section.

Replicates

- You should report how often each experiment was performed
- You should include a definition of biological versus technical replication
- The data obtained should be provided and sufficient information should be provided to indicate the number of independent biological and/or technical replicates
- If you encountered any outliers, you should describe how these were handled
- Criteria for exclusion/inclusion of data should be clearly stated
- High-throughput sequence data should be uploaded before submission, with a private link for reviewers provided (these are available from both GEO and ArrayExpress)

Please outline where this information can be found within the submission (e.g., sections or figure legends), or explain why this information doesn't apply to your submission:

Criteria for exclusion of participants are explicitly reported in the 'Participants and design' section. Data of 5 participants had to be excluded from the EEG analysis because of technical failure during the EEG measurement. This information can be found in the "Statistical analysis" section. For the MVPA decoding during the learning task, we only included participants with a statistically significant classification accuracy during the classifier training (i.e., we excluded participants for the decoding, if it was not possible to train a classifier that discriminates categories significantly above chance performance). Importantly, however, we additionally computed all analyses again including all participants regardless of significant classification scores during the classifier training. This additional analysis left our findings largely unchanged. Results of this additional analysis are reported in the "Supplementary Results" section.

Statistical reporting

- Statistical analysis methods should be described and justified
- Raw data should be presented in figures whenever informative to do so (typically when N per group is less than 10)
- For each experiment, you should identify the statistical tests used, exact values of N, definitions of center, methods of multiple test correction, and dispersion and precision measures (e.g., mean, median, SD, SEM, confidence intervals; and, for the major substantive results, a measure of effect size (e.g., Pearson's r, Cohen's d)
- Report exact p-values wherever possible alongside the summary statistics and 95% confidence intervals. These should be reported for all key questions and not only when the p-value is less than 0.05.

Please outline where this information can be found within the submission (e.g., sections or figure legends), or explain why this information doesn't apply to your submission:

The statistical analysis methods are specified in the sections 'Statistical analysis', 'EPP analysis', 'MVPA training and 'MVPA decoding'. The relevant statistics are reported in the 'Results' section.

(For large datasets, or papers with a very large number of statistical tests, you may upload a single table file with tests, Ns, etc., with reference to sections in the manuscript.)

Group allocation

- Indicate how samples were allocated into experimental groups (in the case of clinical studies, please specify allocation to treatment method); if randomization was used, please also state if restricted randomization was applied
- Indicate if masking was used during group allocation, data collection and/or data analysis

Please outline where this information can be found within the submission (e.g., sections or figure legends), or explain why this information doesn't apply to your submission:

Participants were randomly assigned to experimental groups. This information can be found in the 'Participants and design' section.

Additional data files ("source data")

- We encourage you to upload relevant additional data files, such as numerical data that are represented as a graph in a figure, or as a summary table
- Where provided, these should be in the most useful format, and they can be uploaded as "Source data" files linked to a main figure or table
- Include model definition files including the full list of parameters used
- Include code used for data analysis (e.g., R, MatLab)
- Avoid stating that data files are "available upon request"

Please indicate the figures or tables for which source data files have been provided:

Data and code reported in this manuscript are made publicly available in the corresponding author's GitHub repository, as explicitly stated in the 'Data and code availability' section.
